# Cross-cultural validation and analysis of responsiveness of the QUALIOST^®^: QUAlity of Life questionnaire In OSTeoporosis

**DOI:** 10.1186/1477-7525-3-69

**Published:** 2005-11-10

**Authors:** Christine de la Loge, Kate Sullivan, Robert Pinkney, Patrick Marquis, Christian Roux, Pierre Jean Meunier

**Affiliations:** 1 Mapi Values, 27 rue de la Villette, 69003, Lyon, France; 2 Mapi Values, 15 Court Square Suite 620, Boston, MA 02108, USA; 3 CEMO, Hôpital Cochin, Service de Rhumatologie, 27 rue du Faubourg Saint-Jacques, 75679, Paris Cedex 14, France; 4 Unité INSERM 403, Faculté Laennec, Rue Guillaume Paradin, 69372, Lyon Cedex 8, France

## Abstract

**Background:**

The QUALIOST^® ^was designed for use with the SF-36 to measure established osteoporosis-specific quality of life (QoL). The reliability (internal consistency and test-retest) and validity of the questionnaire were established in a stand-alone psychometric validation study. The objective of this paper is to provide additional information on the instrument's responsiveness using clinical trial data, along with the reliability and validity of translated versions.

**Methods:**

The Spinal Osteoporosis Therapeutic Intervention (SOTI) was an international clinical trial comparing strontium ranelate to placebo on the occurrence of new vertebral fracture in patients with postmenopausal osteoporosis. QoL was a secondary endpoint, assessed using the SF-36 and QUALIOST^® ^at baseline and every six months, with the main analysis at 3-year follow-up. Questionnaire acceptability, analysis of the hypothesised structure, internal consistency reliability and responsiveness to clinical change over time were assessed at the 3-year follow up.

**Results:**

1592 patients from 11 countries completed at least one QoL questionnaire. The psychometric properties of the questionnaires were assessed on cross-sectional (N = 1486) and longitudinal (N = 1288) data. Item discriminant validity of the QUALIOST^® ^was excellent, as was item convergent validity, with 100% of item-scale correlations being above the 0.40 level. Internal consistency reliability was also extremely good, with high Cronbach's alpha scores above the 0.70 benchmark. Responsiveness results were consistent for all QUALIOST^® ^scores, indicating that greater decreases in QoL corresponded to greater numbers of fractures experienced. QUALIOST^® ^scores also differed according to the type of fracture suffered. This was demonstrated by increased effect sizes for more severe vertebral fractures (clinical vertebral and painful vertebral). In comparing responsiveness, the QUALIOST^® ^scores were generally more consistent than those of the SF-36. Most notably, the QUALIOST^® ^was more responsive with regard to painful vertebral fractures than the SF-36.

**Conclusion:**

The QUALIOST^® ^is a reliable and valid tool for measuring QoL in postmenopausal osteoporotic women. Being available in several validated language versions, it is ready to be used in a variety of settings, including international clinical trials.

## Background

Osteoporosis is a debilitating chronic disease that can reduce quality of life (QoL) in a variety of ways, including diminished physical and emotional functioning. Experiencing fractures can lead to reduced mobility and may be very painful, which can limit everyday activities. Reduced activities can lead to increasing isolation, which negatively impacts upon self-esteem and self-image, and causes depression. Experiencing a vertebral fracture can result in fear of future vertebral fractures and anxiety is reported early in osteoporosis [1], which often also leads to reduced activities.

In 1995, a clinical development program for a new chemical entity, strontium ranelate, was implemented in postmenopausal women with osteoporosis. The SOTI (Spinal Osteoporosis Therapeutic Intervention) study was an international clinical trial comparing strontium ranelate to placebo, according to the double blind procedure, on the occurrence of new vertebral fractures in postmenopausal patients with established osteoporosis. All patients were included after giving informed consent and the protocol was approved by the Ethics Committee. Baseline and annual X-rays were provided and assessed by a centralised procedure (Pr C. Roux, Cochin Hospital, France). The prevalent and incident vertebral fractures were diagnosed using the semi-quantitative method (HK Genant [2]), a visual radiographic approach with specified fracture definitions, routinely used in clinical studies.

QoL was a secondary endpoint, assessed at baseline and every six months, with the main analysis at the three-year follow-up. 1592 patients from 11 countries completed at least one QoL questionnaire. As part of this study, a specific quality of life measure was designed. At this time, there was a need for a short, reliable, valid and responsive instrument to measure the impact of the disease on patients' QoL, which would be available in several different languages for use in international clinical trials. The QoL instruments available at the time did not meet all of these criteria. The Osteoporosis Quality of Life questionnaire (OQLQ) [3] in its first version had 168 questions and the Osteoporosis Functional Disability questionnaire (OFDQ) [4] focused mainly on pain and handicap rather than QoL and was not generated using patient interviews. Based on the lack of availability of a suitable instrument, it was decided to develop the QUALIOST^®^, a QoL questionnaire specific to osteoporosis. Questionnaire acceptability, analysis of the hypothesised structure, internal consistency reliability and responsiveness to clinical change over time were assessed on baseline cross-sectional (N = 1486) and longitudinal data (N = 1288).

The QUALIOST^® ^was developed as an additional module to supplement the SF-36 generic questionnaire. The modular approach was chosen to focus on domains that were not already covered by the generic instrument and therefore minimise patient burden. It focuses mainly on the impact of vertebral fractures on QoL. The SF-36 was identified as the most appropriate generic instrument for this purpose because it is short, validated, and already available in many languages and has content which is relevant to the condition.

The first stage in the development of the QUALIOST^® ^consisted of identifying relevant concepts by conducting interviews and focus group meetings with patients in France and the UK. A back-translation of the English version was conducted following standard procedures [5] to ensure consistency in the final versions.

The next step in the development was an independent validation study to establish the psychometric properties of the QUALIOST^®^. Internal consistency and test-retest reliability, as well as analysis of the hypothesised structure, concurrent and clinical validity of the questionnaire were established in both languages in a population of women with postmenopausal osteoporosis [6].

Following the successful psychometric validation of the QUALIOST^®^, the next task was to assess the responsiveness (the ability of the questionnaire to detect a change in QoL when a fracture occurs) of the instrument in a clinical trial setting and validate additional language versions. This was done during the SOTI study, which evaluated the efficacy of strontium ranelate compared to placebo, on the incidence of new vertebral fracture in an international population (N = 1649) of postmenopausal osteoporotic women. A significant 41% reduction in the relative risk of experiencing a first new vertebral fracture (semi-quantitative assessment according to HK Genant [2]) was observed with strontium ranelate over the three-year study compared to placebo [7]. In this paper, assessment of the validation of the hypothesised structure, internal consistency reliability, and responsiveness to clinical change are presented. The data from 7 countries, for which at least 80 patients had a baseline completed questionnaire, were analysed to validate the different linguistic versions of the QUALIOST^® ^and to confirm the relevance of analysing pooled data.

## Methods

The Spinal Osteoporosis Therapeutic Intervention (SOTI) study was a 12-country, double blind, randomised, controlled trial, with two parallel groups of 2 g orally per day of strontium ranelate versus placebo. Women were eligible for the study if they were at least 50 years old, had been postmenopausal for at least five years, had experienced at least one previous vertebral fracture and had a lumbar bone mineral density less than or equal to 0.840 g/cm^2 ^(Hologic). Study duration was five years with the main statistical analysis planned after three years. 1649 patients were included (1592 in the QoL population).

The primary endpoint was the incidence of patients with a first new vertebral fracture over 3 years. Vertebral fractures were diagnosed by the semi-quantitative method (HK Genant [2]), a visual radiographic approach which corresponds to the attribution of grades (ranging from 0 (no vertebral fracture), 1 (20% decrease of vertebra height), 2 (between 20 and 40% decrease of vertebra height) to 3 (severe vertebral fracture, more than 40% decrease of vertebra height)). One of the secondary endpoints of the clinical trial was the change in QoL, which was assessed at baseline (M0) and then every six months using the SF-36 and QUALIOST^®^. The 12 countries of the SOTI trial were Australia, Belgium, Denmark, France, Germany, Greece, Hungary, Italy, Poland, Spain, Switzerland and the UK. QoL was studied in all countries except Greece, where no validated SF-36 questionnaire existed at the time of study commencement.

The QUALIOST^® ^is a 23 item questionnaire specific to osteoporosis, focussing on vertebral fractures and measures QoL over the previous four weeks (for more details see additional file 1: QUALIOST^® ^Items). The QUALIOST^® ^was developed to be used in conjunction with the SF-36. The items are numbered from 12 to 34, to follow on from the SF-36 (numbered 1 to 11). It includes two dimensions: Physical (10 items) and Emotional (13 items). Scores for each dimension as well as a Total score can be calculated by summing the items and then transforming the sum into a score from 0 to 100 [6], where 100 indicates the highest impairment and 0 the lowest impairment of QoL. If more than half of the items in a dimension are missing then the score is considered missing for that dimension. A Total score is only calculated if both dimension scores are present. The QUALIOST^® ^was originally developed in UK English and French. It was translated following standard forward-backwards techniques [5] and is now also available in Danish, Dutch, Flemish, Belgian French, Australian English, German, Austrian German, Hungarian, Italian, Polish, and Spanish.

The SF-36 measures 8 multi-item dimensions (Physical Functioning [PF], Role-Physical [RP], Bodily Pain [BP], General Health perceptions [GH], Vitality [VT], Role-Emotional [RE], Social Functioning [SF], Mental Health [MH]) and provides two summary scores (Mental Component Summary score = MCS; and Physical Component Summary score = PCS). In addition, an item measures health transition. The recall period is four weeks. Scores were calculated as recommended by the authors [8,9]. For all scores, high values indicate good QoL and low values indicate poor QoL. The SF-36 has been thoroughly validated and used with many different diseases such as hypertension, diabetes, congestive heart failure and cancer [8,9].

Questionnaire acceptability was assessed by considering rates of missing data both in terms of missing questionnaires per visit and missing items per questionnaire. This was assessed for each country and for overall pooled data (combined for all countries) for both questionnaires.

The following analyses were considered at country level, to assess whether the different language versions had similar psychometric properties, so that data could be pooled between countries. As some countries had few patients, they were not analysed individually, but were included in the overall pool. Some analyses varied from this procedure, as outlined below.

The psychometric properties of both the QUALIOST^® ^and SF-36 were assessed on baseline cross-sectional (N = 1486) and longitudinal data (N = 1288).

Validation of the hypothesised structure of the QUALIOST^® ^was assessed at individual country level (in countries with at least 80 patients), and on pooled data. This was performed using multitrait analysis [10] to measure item discriminant validity (items should have a higher correlation with their own dimension rather than with other dimensions) and item convergent validity (each item should be correlated with their own dimension at or above 0.40). Evaluation of floor and ceiling levels were performed to ensure that the questionnaire had the potential to capture an improvement or deterioration in each patient (floor and ceiling levels refer to the percentage of respondents having the respective lowest or highest possible score). For the QUALIOST^®^, a high floor (and respectively a high ceiling) level would imply that the questionnaire would not be capable of measuring an improvement (respectively a deterioration) in QoL. Internal consistency reliability consists of measuring the extent to which individual items are consistent with each other and reflect a single underlying construct, and was assessed by calculating Cronbach's alpha values, with a value of 0.7 or greater being considered as evidence of good reliability [11]. Internal consistency reliability was assessed at country level for countries with at least 80 patients, and on overall pooled data.

Responsiveness to change over time evaluates the ability of the questionnaire to detect changes in clinical status, in this case measuring changes in QoL linked to osteoporotic fracture occurrence in osteoporosis. Groups of patients were defined according to the occurrence or not of the following types of fracture: all types of osteoporotic fracture (vertebral and non vertebral fractures), vertebral fracture, clinical vertebral fracture (defined as a vertebral fracture which occurs with pain and/or with body height loss of ≥ 1 centimetre), painful vertebral fracture (defined as vertebral fracture with pain) and according to the total number of osteoporotic fractures for the categories of all types of osteoporotic fracture.

For each group, the changes in scores between baseline and the last evaluable questionnaire were described, with paired t-tests being performed to assess whether the change was statistically significant. In addition, a one-way analysis of variance was used to compare the mean change in QUALIOST^® ^total score according to the number of fractures (0; 1; 2; ≥ 3) that occurred. Effect sizes (ES) are usually calculated to interpret the sensitivity of scores to clinical change [12]. ES were calculated by subtracting the mean score at baseline from the mean score at endpoint (last evaluable questionnaire) and dividing by the standard deviation of the change between baseline and endpoint. ES were interpreted as small (0.20), medium (0.50) or large (0.80) [13]. It was expected that if a woman had a fracture during the study, she would rate a lower QoL at endpoint compared to baseline, expressed as a decrease in scores for the SF-36 and as an increase in scores for the QUALIOST^®^.

Responsiveness of the QUALIOST^® ^was assessed on pooled data only; individual country analysis was not possible due to the low number of patients who experienced fractures at country level.

## Results

This study was completed in June 2003. The data for the main 3-year analysis were collected between November 1996 (first inclusion) and June 2001. A total of 1592 patients completed at least one QoL questionnaire (QUALIOST^® ^or SF-36 at least once during the study) (representing 96.5% of the patients included in the trial). A total of 1486 patients were included in the analysis of cross-sectional data and 1288 in the analysis of longitudinal data on the psychometric properties of the QUALIOST^® ^and SF-36.

At baseline, patients were, on average, 70 years old and the majority were living in their own home (95.7%). The mean Body Mass Index was 26.2 (SD ± 4.1). Following inclusion, a central reading centre confirmed that 90.2% of patients had at least one prevalent osteoporotic fracture, and 87.3% of patients had at least one vertebral fracture, as determined by semi-quantitative methods.

A rapid and sustained vertebral anti-fracture efficacy of strontium ranelate has been demonstrated in the intent-to-treat population, with a relative risk reduction for vertebral fracture of 49% (p < 0.001) in the first year of treatment and 41% (p < 0.001) over 3 years. A significant reduction of the relative risk of multiple vertebral fractures by 36% (p = 0.02) has also been shown. There was a significant increase in lumbar bone mineral density of 14.4% in the strontium ranelate group compared to the placebo group (p < 0.001) and an increase of femoral neck BMD of 8.3% (p < 0.001) over 3 years [14]. Evaluable (less than 50% of missing data) baseline QoL questionnaires were available for 93.3% of patients and were therefore included in the analyses of the hypothesised structure and internal consistency reliability. Longitudinal data were available for 80.9% of the patients for the responsiveness analysis. Most of the patients completed the questionnaire alone (70.9% at baseline) and in the waiting room at the medical centre (64.6% at baseline) rather than at home.

### Quality of completion

There was a high return rate of questionnaires (93.5% at baseline) and quality of completion was high for both questionnaires: 89.3% of baseline QUALIOST^® ^and 76.1% of baseline SF-36 had no missing data; the mean number of items missing per baseline questionnaire was 1.24% (SD ± 7.07) for the QUALIOST^® ^and 2.12% (SD ± 6.55) for the SF-36, indicating particularly high acceptability of the QUALIOST^® ^instrument. By country, the percentage of baseline questionnaires with no missing data ranged between 84.1% in France and 93.6% in Spain for the QUALIOST^®^, and between 68.9% in France and 83.5% in Spain for the SF-36.

### Validation of the hypothesised structure of the QUALIOST^® ^(see Table 1)

Analysis of the cross-sectional psychometric properties by country included validation of the questionnaires structure and internal consistency reliability for the 7 countries that had at least 80 patients: Australia, Belgium, France, Italy, Poland, Spain and the UK. Table 1 provides a summary of results for these individual countries, and overall (11 countries pooled).

**Table 1:** Summary of cross-sectional psychometric properties of the QUALIOST^® ^by country

Scale	Country	N	Item convergent validity	Item discriminant validity	Cronbach's α	Floor (%)	Ceiling (%)
			Range of correlations	Success rate (%)			
Physical	Australia	88	0.49–0.79	90.0	0.91	5.7	0.0
	Belgium	179	0.63–0.79	100.0	0.93	1.1	0.0
	France	148	0.65–0.81	90.0	0.93	0.7	0.0
	Italy	360	0.56–0.70	90.0	0.89	0.6	0.0
	Poland	257	0.53–0.73	90.0	0.90	0.4	0.0
	Spain	107	0.48–0.79	80.0	0.89	0.0	0.0
	The UK	151	0.68–0.87	80.0	0.95	1.3	0.0
	
	Overall	1465	0.62–0.74	90.0	0.92	1.2	0.0

Emotional	Australia	88	0.56–0.75	76.9	0.93	2.3	0.0
	Belgium	179	0.47–0.78	84.6	0.92	0.0	0.0
	France	148	0.58–0.82	100.0	0.93	0.0	0.0
	Italy	360	0.55–0.74	92.3	0.92	0.0	0.0
	Poland	257	0.57–0.76	92.3	0.92	0.0	0.8
	Spain	107	0.47–0.81	61.5	0.93	0.0	0.0
	The UK	151	0.61–0.81	84.6	0.95	0.7	0.0
	
	Overall	1465	0.52–0.78	100.0	0.93	0.3	0.1

Total	Australia	88	0.50–0.85	.	0.95	2.3	0.0
	Belgium	179	0.45–0.79	.	0.95	0.0	0.0
	France	148	0.59–0.80	.	0.96	0.0	0.0
	Italy	360	0.55–0.75	.	0.94	0.0	0.0
	Poland	257	0.52–0.80	.	0.95	0.0	0.0
	Spain	107	0.45–0.85	.	0.95	0.0	0.0
	The UK	151	0.59–0.87	.	0.97	0.7	0.0
	
	Overall	1465	0.56–0.80	.	0.95	0.3	0.0

Item convergent validity success rate: 100% for each scale (all countries)

The item convergent validity showed excellent scaling results for the pooled data with all item scale-correlations above 0.40. Similar scaling success was found in all country versions of the QUALIOST^®^. The item discriminant validity was good for both dimensions (physical and emotional). For the pooled data all items except one had the highest correlation with their own dimension (the exception was Q30: "During the past 4 weeks, has osteoporosis been a daily problem?" which correlated with its own Physical score at 0.70, and with the Emotional score at 0.78). At country level, there were slight variations but for most countries there was only one or two items not meeting the criterion. The item most frequently correlated with the competing score was Q30 (in all countries except Belgium). Together, the item convergent validity and item discriminant validity results demonstrated the satisfactory validity of the QUALIOST^® ^hypothesised structure for each language version tested (7 countries) and for overall data (11 countries) (see Table 1).

### Internal consistency reliability of the QUALIOST^® ^(see Table 1) and distribution of baseline scores

The results of the internal consistency reliability were excellent in all countries and for pooled data, with α values ranging from 0.89 to 0.95 for the Physical dimension (0.92 pooled data), from 0.92 to 0.95 (0.93 pooled data) for the Emotional dimension, and 0.94 to 0.97 for the Total score (0.95 pooled data).

The absence of major floor or ceiling effects indicated that the questionnaire had the potential to capture an improvement or deterioration in QoL for the analysed population. The greatest percentage of respondents at floor or ceiling for any of the QUALIOST^® ^scores was 5.7% (Australia: physical dimension).

The distribution of baseline QUALIOST^® ^scores is provided in Table 2.

**Table 2:** Baseline QUALIOST^® ^scores according to number of osteoporotic fractures (all types) experienced

Score	No of fractures	Frequency	Mean	SD	ANOVA (p-value)
Physical	0	839	36.91	22.72	0.2330
	1	274	39.94	22.63	
	2	81	37.84	21.65	
	> = 3	77	39.78	24.35	
Psychological	0	840	40.07	23.07	0.1255
	1	274	43.65	22.02	
	2	81	43.02	22.34	
	> = 3	77	41.00	22.76	
Total	0	837	38.63	21.63	0.1341
	1	274	42.04	20.92	
	2	81	40.77	21.15	
	> = 3	77	40.47	22.15	

Baseline scores based on number of fractures occurring between baseline and endpoint

### Validity and reliability of the SF-36

It is usually recommended to confirm the psychometric properties of an instrument whenever it is used in a new population [15]. Therefore, the psychometric properties of the SF-36 were assessed in the current study population. However, as the SF-36 is already validated [8,9], details of the hypothesised structure analysis and internal consistency reliability are not presented here. The item convergent validity for the SF-36 showed that overall, 97% of item-scale correlations were greater than or equal to 0.40. At country level, some items were below 0.40, for example Q11c: "I expect my health to get worse" (6 out of 7 countries). The item discriminant validity of the SF-36 showed 100% scaling success for all dimensions on the pooled data. At country level, some items did not meet the criterion, notably in the general health perceptions dimension. Internal consistency reliability of the SF-36 was good, with all Cronbach's alpha values for pooled data being above 0.70 (range 0.74–0.89). At country level, Poland and Italy were below 0.7 for the general health perceptions dimension, as was the social functioning dimension in Poland.

### Responsiveness of the QUALIOST^®^

The analysis of responsiveness showed consistent results for all 3 QUALIOST^® ^scores, assessed according to the number of vertebral and non-vertebral fractures that occurred during the study. Responsiveness indicated a greater decrease in QoL in patients with more fractures; mean change in QUALIOST^® ^scores for 0, 1, 2 and 3 or more fractures can be seen in Table 3.

**Table 3:** Mean change in QUALIOST^® ^scores according to the number of osteoporotic fractures (all types) experienced

No of fracture		0	1	2	≥3
Physical	N	836	272	81	77
	Mean (± 95% CI)	-0.31 (± 1.38)	1.21 (± 2.48)	5.18 (± 4.09)	6.54 (± 4.84)
	Effect Size	-0.02	0.06	0.28	0.30
Emotional	N	838	274	81	77
	Mean (± 95% CI)	-1.55 (± 1.33)	-0.01 (± 2.29)	3.83 (± 3.98)	5.57 (± 3.65)
	Effect Size	-0.08	-0.00	0.21	0.34
Total	N	832	272	81	77
	Mean (± 95% CI)	-0.92 (± 1.25)	0.46 (± 2.18)	4.41 (± 3.73)	5.99 (± 3.72)
	Effect Size	-0.05	0.02	0.26	0.36

Mean change measured between baseline and endpoint

ES = (mean at endpoint – mean at baseline)/standard deviation of change

ES for the number of fractures also demonstrated a clear trend of greater change with more fractures. The ES for the number of fractures are presented in Table 3. For 3 or more fractures the range across scores was 0.30–0.36, indicating a small change. The mean change in scores reached significance in the Physical dimension for those experiencing 2 or > = 3 fractures (p = 0.0150 and p = 0.0098 respectively) using a paired t-test. In the Emotional dimension the mean change in scores reached significance for those experiencing > = 3 fractures (p = 0.0038). The Total QUALIOST^® ^score indicated a significant mean change from 0 for those suffering 2 fractures (p = 0.0228) and those experiencing > = 3 fractures (p = 0.0023). The difference in mean change between the groups experiencing different numbers of fractures reached significance in the Physical (p = 0.0062), Emotional (p = 0.0026) and Total (p = 0.0016) scores using an ANOVA.

The QUALIOST^® ^scores also increased according to the type of vertebral fracture, as demonstrated by mean change in scores and increased ES (summarised in Table 4). For more severe vertebral fractures in terms of symptoms, the responsiveness increased with the occurrence of fractures in the following order: vertebral, clinical vertebral, painful vertebral. For osteoporotic fractures of any type, the ES was lower than for vertebral fractures. If patients had a fracture, they tended to have a positive mean change in score, indicating a decrease in QoL and patients without a fracture had a slight decrease in their scores, indicating a small improvement in their QoL. ES for painful vertebral fractures ranged from 0.46 to 0.61 across scores, indicating a medium change.

**Table 4:** Mean change in QUALIOST^® ^scores according to type of fracture

Type of fracture	Physical		Emotional		Total	
	Mean (± 95% CI)	Effect Size	Mean (± 95% CI)	Effect Size	Mean (± 95% CI)	Effect Size
Osteoporotic (N = 429)	2.99 (± 1.96)	0.14	1.70 (± 1.77)	0.09	2.22 (± 1.70)	0.12
Vertebral (N = 315)	3.59 (± 2.34)	0.17	2.15 (± 2.09)	0.11	2.73 (± 2.01)	0.15
Clinical vertebral (N = 169)	4.90 (± 3.39)	0.22	3.91 (± 2.96)	0.20	4.34 (± 2.91)	0.22
Painful vertebral (N = 89)	5.84 (± 4.43)	0.61	5.62 (± 3.81)	0.46	5.71 (± 3.79)	0.59

Mean change measured between baseline and endpoint

ES = (mean at endpoint – mean at baseline)/standard deviation of change

### Comparison of responsiveness of the QUALIOST^® ^and SF-36

There were some differences in the responsiveness of the two questionnaires. The QUALIOST^® ^scores indicated deterioration in QoL for patients with a fracture and a very slight improvement in QoL for patients without a fracture. Generally, ES for the QUALIOST^® ^were very consistent. For those experiencing a fracture, they tended to increase by type of fracture with the smallest ES being for those experiencing osteoporotic fractures (ES range of 0.09 to 0.14) and the largest ES being observed for those experiencing painful vertebral fractures (range of 0.46 to 0.61). ES for the QUALIOST^® ^scores according to the 4 categories of fracture are displayed in Table 4.

The SF-36 scores indicated deterioration in QoL for both groups, with patients who had a fracture experiencing greater deterioration. The PF dimension demonstrated itself to be most responsive in the SF-36 (ES range of -0.26 to -0.41), with the GH dimension also being notably responsive (ES range of -0.23 to -0.38). ES for the SF-36 scores according to the 4 categories of fracture are displayed in Table 5.

**Table 5:** Effect sizes for change in SF-36 scores according to type of fracture

Type of fracture	PF	RP	BP	GH	VT	SF	RE	MH	PCS	MCS
Osteoporotic (n = 436)	-0.27	-0.14	-0.08	-0.25	-0.05	-0.25	-0.16	-0.15	-0.18	-0.18
Vertebral (n = 318)	-0.26	-0.12	-0.11	-0.23	-0.07	-0.27	-0.15	-0.16	-0.17	-0.19
Clinical vertebral (n = 170)	-0.33	-0.11	-0.09	-0.28	-0.14	-0.27	-0.17	-0.17	-0.23	-0.18
Painful vertebral (n = 89)	-0.41	-0.17	-0.18	-0.38	-0.22	-0.30	-0.21	-0.30	-0.30	-0.24

Change in SF-36 scores measured between baseline and endpoint: effect sizes only presented for those experiencing a fracture

ES = (mean at endpoint – mean at baseline)/standard deviation of change

## Discussion

Given the length of follow-up (3 years), the high return rates of the QUALIOST^® ^and low percentages of missing data, this study demonstrated good acceptability of the questionnaire. Rates of questionnaires with at least one missing data element varied slightly between countries but gave no cause for concern in any particular country.

Analyses of the hypothesised structure and internal consistency reliability in the 7 countries with at least 80 participants demonstrated that each of these language versions had satisfactory psychometric properties. Item convergent validity was excellent with all items of all versions reaching 100% scaling success. Item discriminant validity showed the items to be highly consistent. Ten items correlated higher with the competing scale, although most of the items not meeting the criterion were highly correlated with their own score and only slightly higher with the competing score. The close correlations between dimension scores for these items reflects the relatively high correlations between the two dimensions overall, which is required when computing a global score. The Emotional score results were slightly weaker than the other two scores, the weakest result being in Spain with 5 items not meeting the item discriminant validity criterion. However, as only one item of the Emotional score produced a significantly different correlation, the results were considered to be satisfactory. Cronbach's alpha values being above the 0.70 threshold for all versions and scales indicated excellent internal consistency reliability, and no major floor or ceiling effects were observed for any score. Altogether, these results led to confidence in being able to analyse pooled data.

The analysis of responsiveness to clinical change showed a consistent link between both the occurrence and number of fractures with change in QUALIOST^® ^scores. ES showed greater responsiveness for sub-categories of increasing severity in vertebral fracture according to the following order: vertebral, clinical vertebral and painful vertebral fractures. Although results were consistent, with higher responsiveness obtained for increasing severity in vertebral fractures, the ES were rather low, except for painful vertebral fractures. For fractures of any type, the low ES can partly be explained by the fact that responsiveness was assessed using the last completed questionnaire, and not using a questionnaire completed immediately after the occurrence of fractures, leaving time for at least partial recovery. Clearly, if responsiveness had been assessed using QoL data gathered just after the occurrence of fractures, then larger mean changes and ES would have been obtained.

Generally, when looking at responsiveness, the ES of the QUALIOST^®^scores tended to be more consistent than those of the SF-36. Most notably, the QUALIOST^® ^demonstrated higher responsiveness than the SF-36 for painful vertebral fractures. The GH dimension of the SF-36 was highly responsive, demonstrating the importance in measuring the global impact that fractures had upon patients. Consistently higher ES were observed in the PF dimension of the SF-36 in comparison to the Physical component of the QUALIOST^®^, with the exception of painful vertebral fractures. This could indicate that the SF-36 is more responsive with regard to fractures of lower limbs, especially since the PF dimension is largely concerned with walking. Furthermore, the ES for the BP SF-36 dimension were inconsistent and lower than the equivalent QUALIOST^® ^scores, which could potentially indicate the QUALIOST^® ^to be more responsive to vertebral fractures than the SF-36. This could be expected though since the focus of the QUALIOST^® ^is on vertebral fractures. Therefore, it could be suggested that the SF-36 is more responsive to limb fractures and the QUALIOST^®^to vertebral fractures, demonstrating the value of using the QUALIOST^®^in conjunction with the SF-36.

When the decision was taken to create the QUALIOST^®^, no other questionnaires had been published that were short, osteoporosis-specific instruments with good psychometric properties and available in many languages. Currently, there are several QoL instruments specific to osteoporosis, including the OPTQoL (Osteoporosis Targeted Quality of Life), the OPAQ (Osteoporosis Assessment Questionnaire), the QUALEFFO (Quality of life questionnaire of the European Foundation for Osteoporosis) [16], the OQLQ (Osteoporosis Quality of Life Questionnaire) and the OFDQ (Osteoporosis Functional Disability Questionnaire) [17]. The OPTQoL is a cross-sectional instrument that was developed to characterise the burden of osteoporosis in a community and therefore is not aimed at assessing change over time. The OFDQ measure of pain and disability, designed for use in longitudinal intervention trials involving exercise rehabilitation for patients with osteoporotic vertebral fractures, has demonstrated some usefulness for tracking an individual's change over time [4], although it has not been used in clinical trials of medication and focuses on disability rather than QoL. The OQLQ was administered in an osteoporotic population with chronic back pain [3]. The questionnaire was found to be at least as responsive as other instruments when using a global rating of change. The authors acknowledge that this may have limited validity in this population due to the possible inaccuracy of patients estimating their change. Responsiveness of the OQLQ was not based on clinical trial data with the occurrence of fracture.

The QUALEFFO has been used in a large clinical interventional trial (Multiple Outcomes of Raloxifene Evaluation, or the MORE study) and has been able to discriminate between groups of patients with and without incident vertebral fractures (IVFX) [18]. The OPAQ was also used as an outcome in the MORE study [19] and also seems to be responsive to clinical changes; women with incident vertebral fractures had a higher percentage of significant HRQL loss compared with women without incident vertebral fractures in physical function, symptoms, and overall HRQL (all p < 0.05) but not emotional status or social interaction.

As can be seen, there are now several osteoporosis-specific QoL questionnaires available. Notably the OPAQ and the QUALEFFO have demonstrated responsiveness to clinical change. The main issue that has been stated with the QUALEFFO specific questionnaire is that it performs similarly to the Physical summary score of the SF-36 in discriminating between fracture cases and controls [6]. It would be interesting to compare the discriminative properties of the QUALIOST^® ^with those of these instruments in future work.

As the QUALIOST^® ^is designed to be used in conjunction with the SF-36, the benefits of using a generic and specific questionnaire can be attained whilst minimising burden to patients and administrators [17]. Indeed, one of the main strengths of the QUALIOST^® ^compared with other specific questionnaires such as the QUALEFFO is its measurement strategy in being a supplemental specific module complementing the SF-36 [6]. The advantages of choosing the SF-36 include the relevance of the scores relating to osteoporosis, its availability in many languages and extensive use in many settings, which allows comparisons between populations and conditions. It has also established reference values which can be used to compare with healthy populations, and can be adjusted for age effects, which is useful in the target population of elderly osteoporotic patients.

## Conclusion

The analysis results of the hypothesised structure and internal consistency reliability of 7 languages versions of the QUALIOST^®^, using data from the SOTI trial combined with previously reported psychometric validation results confirm the good psychometric properties of this instrument. The QUALIOST^® ^has demonstrated responsiveness to clinical change (occurrence of new osteoporotic fractures, vertebral fractures, clinical vertebral fractures, and painful vertebral fractures). The QUALIOST^® ^is a short, reliable and valid tool to measure QoL in postmenopausal osteoporotic women. Being available in several validated language versions, it is ready to be used in a variety of settings, including international clinical trials.

## Abbreviations

ANOVA Analysis of Variance

BP Bodily Pain (SF-36)

Emotional Emotional (QUALIOST^®^)

ES Effect Size

GH General Health Perceptions (SF-36)

HRQL Health Related Quality of Life

MCS Mental Component Summary (SF-36)

MH Mental Health (SF-36)

OFDQ Osteoporosis Functional Disability Questionnaire

OPAQ Osteoporosis Assessment Questionnaire

OPTQoL Osteoporosis Targeted Quality of Life

OQLQ Osteoporosis Quality of Life Questionnaire

PCS Physical Component Summary (SF-36)

PF Physical Functioning (SF-36)

Physical Physical (QUALIOST^®^)

QoL Quality of Life

QUALEFFO Quality of Life Questionnaire of the European Foundation for Osteoporosis

RE Role Emotional (SF-36)

RP Role Physical (SF-36)

SF Social Functioning (SF-36)

SF-36 Short Form (36 items)

SOTI Spinal Osteoporosis Therapeutic Intervention

VT Vitality (SF-36)

## Authors' contributions

CDL was involved in the supervision of the statistical analysis and revising the document critically for important intellectual content. KS was involved in the drafting of the document. RP was involved in the drafting of the document and revising it critically for important intellectual content. PM, as the author of the QUALIOST^® ^questionnaire, helped in the design of the analysis and the interpretation and was involved in critically revising the important intellectual content of the document. CR was responsible for the assessments of vertebral fractures in the SOTI study (semi-quantitative and quantitative) and as a major clinical expert in the SOTI study, also validated the clinical aspects of the article. PJM is the SOTI coordinator and as a major clinical expert in the SOTI study, also validated the clinical aspects of the article.

## Supplementary Material

Additional File 1 QUALIOST^®^* Items:
A list of the 23 questions taken from the QUALIOST^® ^module.
*QUALIOST^® ^is protected by copyright and international trademark registration, with all rights reserved to SERVIER. Do not use without permission. For information on, or permission to use QUALIOST^®^, please contact the Mapi Research Trust, 27 rue de la Villette 69003 Lyon, FRANCE. Tel: +33 (0) 472 13 65 75 – Email: trust@mapi.fr – Website: Click here for file
