# Supplementary material for: Cross-cultural validation and analysis of responsiveness of the QUALIOST®: QUAlity of Life questionnaire In OSTeoporosis
Source: Health Qual Life Outcomes. 2005 Nov 10;3:69. doi: 10.1186/1477-7525-3-69 (PMC1325267; doi:10.1186/1477-7525-3-69)
Supplement: Additional File 1 — QUALIOST®* Items: A list of the 23 questions taken from the QUALIOST® module. *QUALIOST® is protected by copyright and international trademark registration, with all rights reserved to SERVIER. Do not use without permission. For information on, or permission to use QUALIOST®, please contact the Mapi Research Trust, 27 rue de la Villette 69003 Lyon, FRANCE. Tel: +33 (0) 472 13 65 75 – Email: trust@mapi.fr – Website: [file 1477-7525-3-69-S1.doc]

**QUALIOST®* Items:** A list of the 23 questions taken from the QUALIOST® module.

1. Have you been restricted in going out (for a walk, shopping, etc) or travelling?
2. Have you been restricted in your housework (vacuuming, ironing, washing the floor, making the bed…)?
3. Have you been restricted in your hobbies ?
4. Have you had any difficulty getting dressed?
5. Have you had difficulty getting up from a chair or a bed?
6. Have you had pain in the middle or upper part of your back?
7. Have you had pain when walking or climbing stairs?
8. Have you experienced discomfort when staying in the same position for a long time (sitting, standing up)?
9. Has pain interfered with your sleep ?
10. Do you feel older?
11. Have you been affected by a change in your physical appearance (loss in height, shape of your back)?
12. Have you felt less self-confident?
13. Have you felt frustrated?
14. Have you felt worried?
15. Have you been afraid of falling or of having fractures?
16. Have you been worried about not being comfortably seated?
17. Have you had to think about everything you were doing?
18. In general would you say your quality of life has been:
19. Has osteoporosis been a daily problem?
20. Have you felt handicapped because of you osteoporosis?
21. Have you had the feeling you were a burden on others because of your osteoporosis?
22. Have you been afraid of your osteoporosis getting worse?
23. Have you worried about losing your independence because of your osteoporosis?

***QUALIOST® is protected by copyright and international trademark registration, with all rights reserved to SERVIER. Do not use without permission. For information on, or permission to use QUALIOST®, please contact the Mapi Research Trust, 27 rue de la Villette 69003 Lyon, FRANCE. Tel: +33 (0) 472 13 65 75 - Email: trust@mapi.fr - Website: www.mapi-trust.org**
